# MASLD Management in Spain: A Nationwide Survey of Gastroenterologists Highlighting Gaps in Risk Assessment and Primary Care Coordination

**DOI:** 10.3390/jcm15093259

**Published:** 2026-04-24

**Authors:** Carolina Jiménez-González, Paula Argos Vélez, Lorena Cayón, Ana Belén García-Garrido, Noelia Fontanillas Garmilla, Antonio Cuadrado, Paula Iruzubieta, Javier Crespo

**Affiliations:** 1 Clinical and Translational Research in Digestive Diseases, Gastroenterology and Hepatology Department, Valdecilla Research Institute (IDIVAL), Marqués de Valdecilla University Hospital, 39008 Santander, Spain; 2 Family and Community Medicine, Vargas Health Centre, Primary Care Department of the Cantabrian Health Service, 39011 Santander, Spain; 3 Family and Community Medicine, Bezana Health Center, Department of Medicine and Psychiatry, University of Cantabria IDIVAL, Santa Cruz de Bezana, 39100 Cantabria, Spain; 4 Cantabria Cohort, Valdecilla Research Institute (IDIVAL), Marqués de Valdecilla University Hospital, 39008 Santander, Spain; 5 School of Medicine, University of Cantabria, 39011 Santander, Spain; 6 Medicine AI, 28028 Madrid, Spain

**Keywords:** MASLD, metabolic dysfunction-associated steatotic liver disease, gastroenterologists, clinical practice survey, non-invasive liver assessment, cardiometabolic risk, primary care coordination

## Abstract

**Background**: Metabolic dysfunction-associated steatotic liver disease (MASLD) is the most prevalent chronic liver disease worldwide and a major contributor to the global cardiometabolic burden. Early identification of patients at risk of metabolic dysfunction-associated steatohepatitis (MASH) and advanced fibrosis is essential to prevent liver-related and cardiovascular complications. In Spain, the burden of MASLD is increasing, yet information on routine clinical management by gastroenterologists remains limited. **Methods**: A nationwide cross-sectional online survey was conducted among members of the Spanish Society of Digestive Diseases (SEPD). The questionnaire explored five domains: MASLD knowledge, use of non-invasive biomarkers and imaging, awareness and implementation of clinical guidelines, cardiometabolic and alcohol-related risk assessment, and coordination with primary care. **Results**: A total of 429 specialists responded, 33.1% reported more than 20 years of practice and most worked in public hospitals, including 29.2% in large tertiary centers. Awareness of the MASLD definition was high, and 91.2% identified fibrosis as the main prognostic determinant. Non-invasive fibrosis biomarkers were widely used, whereas steatosis biomarkers were less frequently applied. Elastography was available to 96.1%. Guideline knowledge was reported by 80.4%, although implementation was lower. Cardiovascular risk evaluation varied: 75.1% reported systematic screening. Alcohol consumption was usually assessed. Coordination with primary care was limited: 91.1% expressed concerns regarding physicians’ familiarity with MASLD classification, and only 31.1% reported shared protocols. **Conclusions**: Spanish gastroenterologists show high awareness of MASLD and broad access to non-invasive diagnostic tools. However, important gaps remain in cardiovascular and alcohol risk assessment, guideline implementation, and coordination with primary care.

## 1. Introduction

Metabolic dysfunction-associated steatotic liver disease (MASLD) currently represents the leading cause of chronic liver disease worldwide, with an estimated prevalence of approximately 25–38% of the adult population globally, and it is projected to become one of the leading indications for liver transplantation in the coming decades [1].

In Spain, population-based studies have confirmed the high burden of fatty liver disease, underscoring the magnitude of this condition within the country [2]. The shift in nomenclature towards MASLD reflects the increasing recognition of the metabolic complexity of this condition, which is closely linked to obesity and type 2 diabetes mellitus [3,4]. This paradigm shift highlights that MASLD should be considered the hepatic manifestation of systemic metabolic dysfunction rather than an isolated liver disorder [5]. In this context, recent contributions have highlighted the need for a broader clinical perspective in patients with obesity, in whom digestive manifestations extend beyond weight management alone [6].

The clinical spectrum of MASLD ranges from simple steatosis to metabolic dysfunction-associated steatohepatitis (MASH), with the potential to progress to advanced fibrosis, cirrhosis, and hepatocellular carcinoma. Fibrosis remains the strongest prognostic determinant of both liver-related and overall mortality [7]. Beyond the liver, MASLD is independently associated with increased cardiovascular morbidity and mortality, which constitutes the leading cause of death among affected individuals [5,8].

Its silent course and the lack of specific symptoms contribute to delayed diagnosis [5], representing a missed diagnostic and therapeutic opportunity. This issue has become particularly relevant in the current therapeutic landscape, with the development of disease-modifying treatments targeting metabolic dysfunction-associated steatohepatitis. The phase III trial of resmetirom demonstrated histological improvement in patients with MASH and fibrosis [9], while glucagon-like peptide-1 receptor agonists such as semaglutide have also shown promising results in this population [10].

Several scientific societies have developed clinical practice guidelines recommending structured screening and referral algorithms supported by non-invasive biomarkers and elastography [11,12]. However, as highlighted by international studies such as the POP-NEXT project, a persistent gap remains between guideline recommendations and their implementation in clinical practice, even among specialists [13]. Recently, a Spanish multidisciplinary guideline emphasized the need for an integrated approach to patients with metabolic steatotic liver disease involving gastroenterology, endocrinology, internal medicine, and primary care [14]. Against this background, assessing current clinical practice patterns among gastroenterology specialists is essential to understand the real-world readiness to address MASLD. Identifying gaps in risk stratification, use of non-invasive diagnostic tools, and cross-disciplinary coordination [15] is therefore crucial to guide the development of integrated hepatometabolic care pathways capable of delivering early diagnosis, effective risk stratification, and equitable access to emerging therapies.

## 2. Materials and Methods

Study Design

This was a cross-sectional, observational, descriptive, web-based survey of gastroenterology specialists in Spain. The study adhered to the Strengthening the Reporting of Observational Studies in Epidemiology (STROBE) guidelines for observational studies [16] and the Checklist for Reporting Results of Internet E-Surveys (CHERRIES) [17]. The primary objective was to assess knowledge, use of diagnostic tools, and care coordination in the management of metabolic dysfunction-associated steatotic liver disease (MASLD) among members of the Spanish Society of Digestive Diseases (Sociedad Española de Patología Digestiva, SEPD).

As a self-reported survey, this study is subject to several inherent limitations. Responses may be affected by recall bias and social desirability bias, with participants potentially overreporting adherence to recommended practices. In addition, reported behaviors may not fully reflect actual clinical practice, leading to possible discrepancies between perceived and real-world management. These limitations should be considered when interpreting the results.

Questionnaire development and validation

The questionnaire was developed ad hoc through a two-round Delphi process involving 12 experts (6 hepatologists and 6 gastroenterologists) and was subsequently reviewed by a multidisciplinary committee before approval by the SEPD Executive Board. A pilot test with 30 gastroenterology specialists assessed clarity, comprehensibility, relevance, and estimated completion time, resulting in a final questionnaire comprising 53 items. Internal consistency was acceptable (Cronbach’s α = 0.81).

The questionnaire covered five domains:

1. General knowledge of MASLD.
2. Use of non-invasive biomarkers (serum indices and imaging techniques).
3. Awareness and application of clinical practice guidelines.
4. Assessment of cardiometabolic factors, vascular risk, and alcohol consumption.
5. Cross-level coordination, organizational barriers, and frequency of longitudinal reassessment during follow-up.

The full questionnaire, including all sections and survey items, is provided as Supplementary Material File S1.

Study population and sampling

The survey was distributed by email to all active members of the Spanish Society of Digestive Diseases (SEPD) in 2023 (*n* = 1906) via the REDCap^®^ secure web-based platform. A reminder was issued at four weeks. Participation was voluntary and anonymous. We received a total of 429 questionnaires, corresponding to a response rate of 22.5%. Although a non-probabilistic voluntary sampling strategy was employed, responses were obtained from physicians working across multiple geographic regions and different professional profiles (gastroenterology, hepatology, and endoscopy). Potential response bias towards clinicians with a specific interest in MASLD was acknowledged and considered during data interpretation. Therefore, our findings should be interpreted with caution and may not fully represent the knowledge and practices of all gastroenterologists in Spain.

Data collection

Data collection took place between 1 September and 15 October 2023. Electronic informed consent was obtained from all participants prior to survey initiation. No incentives were offered. Data integrity was ensured through automated input validation and restriction of duplicate submissions through unique survey links within the REDCap^®^ platform. For each survey item, analyses were performed using the number of respondents to that specific question as the denominator. Percentages were calculated accordingly. Multiple responses were allowed for selected questions, as specified in the questionnaire. Missing data were handled using available-case analysis, and no imputation was performed.

Ethical considerations

Participation of healthcare professionals was voluntary and anonymous, and did not include patient data. Participants provided their explicit consent by voluntarily taking part in the survey. Data processing complied with the European Union General Data Protection Regulation (EU 2016/679) and applicable national legislation on data protection.

Statistical analysis

Qualitative variables were summarized as absolute frequencies and percentages, and quantitative variables as means with standard deviations (SD). Comparisons between groups were performed using the χ^2^ test for categorical variables and Student’s *t* test for continuous variables, as appropriate. Analyses were performed on a per-question basis using available-case analysis. Missing data were not imputed, and no sensitivity analyses were conducted. All statistical analyses were conducted using IBM SPSS Statistics version 29.0 (IBM Corp., Armonk, NY, USA). A two-sided *p* value < 0.05 was considered statistically significant.

Artificial intelligence was used to review the grammar of the article.

## 3. Results

### 3.1. Sample Characteristics

A total of 429 questionnaires were reported, corresponding to a 22.5% response rate among invited active members of the Spanish Society of Digestive Diseases (SEPD). Percentages reported in the following analyses were calculated based on the number of respondents to each question; therefore, denominators may vary due to missing responses. Among respondents, 230 (56.4%) were women and 173 (42.4%) men, while sex was not reported by 26 participants (6.1%). The most represented age groups were 30–40 years (36.3%; *n* = 148) and >50 years (31.9%; *n* = 130). Regarding professional experience, 33.1% (*n* = 135) reported more than 20 years of clinical practice, followed by 24.3% (*n* = 99) with less than 5 years of experience. Most respondents worked in public hospitals (79.4%; *n* = 324), compared with 11.8% (*n* = 48) in private centers and 7.8% (*n* = 32) in mixed public–private settings. Hospital size varied, with 29.2% (*n* = 119) practicing in large hospitals (>750 beds), 42.9% (*n* = 175) in medium-sized hospitals (250–750 beds), and 19.1% (*n* = 78) in smaller centers (<250 beds). Geographical representation included physicians from all Spanish regions, with the highest participation from Madrid (22.3%; *n* = 87), Andalucía (13.6%; *n* = 53), and Asturias (7.7%; *n* = 30). Regarding professional focus, 51.3% (*n* = 220) identified primarily as general gastroenterologists, 44.1% (*n* = 189) as hepatologists, and 44.3% (*n* = 190) as endoscopists, as these categories were not mutually exclusive, some respondents reported expertise in more than one area. Data shown in Table 1.

### 3.2. Knowledge and Perception of MASLD

**Figure 1:**
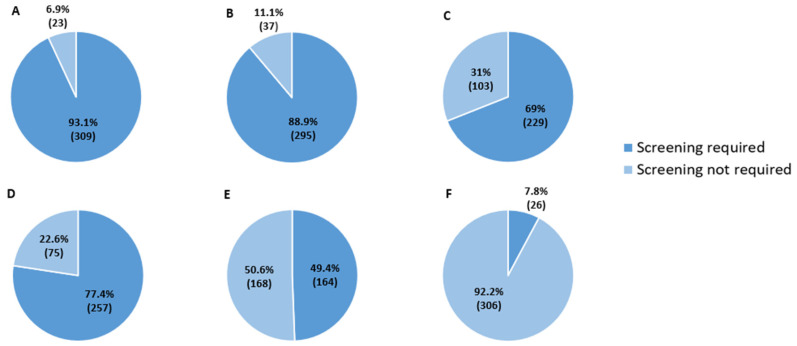
Cases in which the detection of MASLD was considered especially indicated by the respondents. (**A**) Patients with hypertransaminasemia, (**B**) patients with DM2, (**C**) patients with obesity, (**D**) patients with dyslipidemia, (**E**) patients with immune-mediated diseases and (**F**) general population. Each segment corresponds to the percentage of survey responders agreeing with that statement and in brackets the corresponding N. MASLD, metabolic dysfunction-associated steatotic liver disease; DM2, diabetes mellitus II.

Overall, 81.0% (*n* = 264) of respondents reported being familiar with the new MASLD definition, whereas 19.0% (*n* = 62) continued to use the term NAFLD. Among respondents to this question, estimates of hepatic steatosis prevalence varied. In total, 43.2% (*n* = 114) estimated the prevalence between 25% and 50%, while 36.0% (*n* = 95) considered it to be below 25%. Regarding prognostic factors, 91.2% (*n* = 301) correctly identified fibrosis stage as the main determinant of liver-related mortality, compared with 6.4% citing inflammation and 0.6% citing steatosis. As shown in Figure 1, screening for MASLD, in a multiple-choice question, was considered particularly indicated in patients with hypertransaminasemia (93.1%; *n* = 309), type 2 diabetes mellitus (88.9%; *n* = 295), and obesity (BMI > 30 kg/m^2^) (69%; *n* = 229). Only 7.8% (*n* = 26) supported universal population screening.

### 3.3. Clinical Practice and Use of Non-Invasive Biomarkers

**Figure 2:**
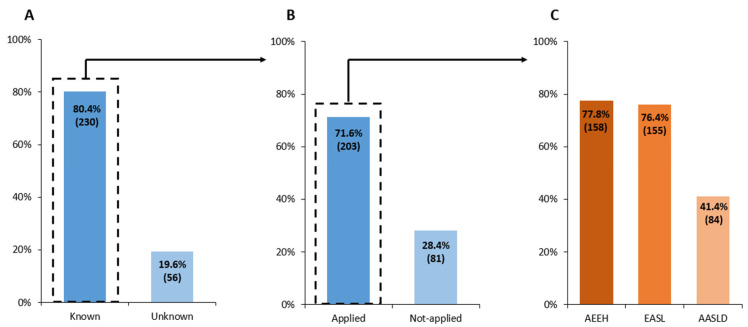
Awareness and application of clinical guidelines. (**A**) Knowledge of clinical practice guidelines, (**B**) their application and (**C**) distribution of the most commonly used guidelines according to the survey. Each bar corresponds to the percentage of survey responders agreeing with that statement and in brackets the corresponding N. AEEH, Spanish Association for the Study of the Liver; EASL, European Association for the Study of the Liver; AASLD, American Association for the Study of Liver Diseases.

Routine use of non-invasive fibrosis biomarkers was reported by 84.9% of respondents (*n* = 346). The most frequently used tools were FIB-4 (63.2%; *n* = 258) and the NAFLD Fibrosis Score (37.5%; *n* = 153), as shown in Table 2. A total of 66.4% (*n* = 271) reported using more than one fibrosis marker in clinical practice. Use of steatosis biomarkers was less frequent. The Fatty Liver Index (FLI) was the most commonly used (22.6%; *n* = 92), followed by the Hepatic Steatosis Index (HSI) (8.8%; *n* = 36) and OWLiver^®^ (5.9%; *n* = 24). However, 39.7% (*n* = 162) reported not using any steatosis biomarker. Regarding imaging techniques, elastography was available to 96.1% of respondents (*n* = 392). The most frequently used modality was FibroScan^®^ (61.0%; *n* = 249), followed by ultrasound (31.4%; *n* = 128) and magnetic resonance imaging (5.6%; *n* = 23). Access to liver biopsy was reported by 93.4% (*n* = 257) of specialists. Awareness of clinical practice guidelines was reported by 80.4% (*n* = 230) of respondents, whereas 71.6% (*n* = 203) indicated that they routinely applied these recommendations in clinical practice. Among those who applied clinical guidelines, the most commonly followed were the Spanish Association for the Study of the Liver (AEEH) guideline (77.8%; *n* = 158) and the European Association for the Study of the Liver (EASL) guideline (76.4%; *n* = 155) (Figure 2).

### 3.4. Cardiometabolic and Alcohol Risk Assessment

Systematic recording of weight, height, and body mass index was reported by 79.7% of respondents (n = 232). However, only 14.1% (*n* = 41) reported regularly measuring waist circumference. Routine cardiovascular risk screening was reported by 75.1% (*n* = 217) of specialists. In contrast, only 17% (*n* = 73) reported evaluating emerging vascular markers such as carotid intima–media thickness, detailed information on the specific risk scores used was not collected. Alcohol intake was assessed by 96.1% of respondents (*n* = 278). However, only 5.6% (*n* = 23) reported using validated screening tools such as the Alcohol Use Disorders Identification Test (AUDIT), while 65% (*n* = 265) relied on non-structured clinical interviews. Additionally, only 19.1% (*n* = 78) reported assessing alcohol intake using biomarkers.

### 3.5. Coordination with Primary Care

**Figure 3:**
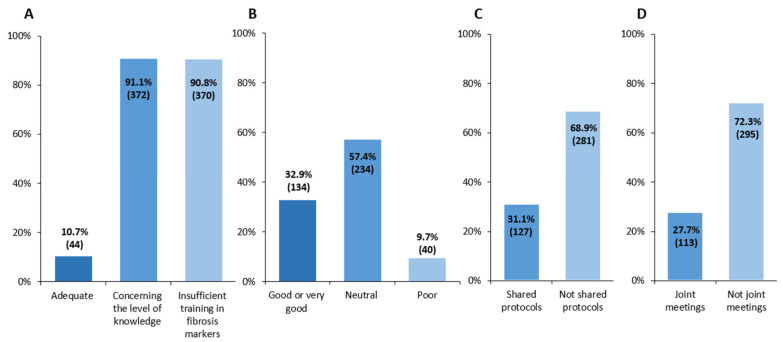
Perceptions of collaboration with primary care and identified barriers. (**A**) Management of MASLD in primary care, (**B**) coordination between specialists and primary care, (**C**) shared protocols between specialists and primary care and (**D**) participation in joint meetings. Each bar corresponds to the percentage of survey responders agreeing with that statement and in brackets the corresponding N. MASLD, metabolic dysfunction-associated steatotic liver disease.

As shown in Figure 3, perceptions of MASLD management in primary care were predominantly negative. In a multiple-choice question, 91.1% (*n* = 372) of respondents expressed concerns regarding primary care physicians’ knowledge of MASLD classification, and 90.8% (*n* = 370) reported limited awareness of fibrosis biomarkers in this setting. Although 68.7% (*n* = 184) acknowledged the availability of ultrasound in primary care, only 15.8% (*n* = 42) reported access to elastography. Regarding alcohol assessment in primary care, 45.9% (*n* = 124) considered current practices inadequate, whereas 33.7% (*n* = 91) indicated uncertainty regarding its adequacy. In terms of collaboration, 57.4% (*n* = 234) described their interaction with primary care as neutral, whereas 32.9% (*n* = 134) considered it good or very good. Most respondents (72.3%; *n* = 295) reported not participating in joint clinical meetings with primary care, and only 31.1% (*n* = 127) reported the existence of shared clinical protocols.

### 3.6. Factors Associated with Clinical Practice

No statistically significant association was observed between years of professional experience and overall knowledge of MASLD management tools. Specialists with more than 10 years of experience and those with less than 5 years of experience reported similar knowledge of steatosis biomarkers (84.2% vs. 87.2%), fibrosis biomarkers (69.5% vs. 64.9%), and clinical guidelines (79.5% vs. 81.4%). However, physicians with less than 5 years of experience reported more frequent use of clinical guidelines than senior specialists (83.3% vs. 68.7%; *p* < 0.05). Hospital size was associated with greater availability of diagnostic tools. In hospitals with more than 750 beds, fibrosis biomarkers and steatosis biomarkers were available in 76.5% and 35.3% of cases, respectively, compared with 44.9% and 11.5% in hospitals with fewer than 250 beds (*p* < 0.001). Use of elastography was also more frequent in larger hospitals (97.2% vs. 86.2%; *p* < 0.05). Differences according to professional focus were also observed. Hepatologists reported higher use of steatosis biomarkers (32.3% vs. 13.9%; *p* < 0.001) and clinical guidelines (89.5% vs. 58.6%; *p* < 0.001) compared with general gastroenterologists. No statistically significant difference was observed in the use of elastography between hepatologists and general gastroenterologists, although a higher use was reported among hepatologists (97.5% vs. 94.1%).

## 4. Discussion

This study provides one of the first nationwide characterizations of the clinical management of metabolic dysfunction-associated steatotic liver disease (MASLD) among gastroenterology specialists in Spain. The results show a high level of awareness regarding the new disease nomenclature and the prognostic relevance of fibrosis, together with widespread use of non-invasive fibrosis biomarkers and elastography. However, important shortcomings in routine clinical practice were identified, including heterogeneity in guideline implementation, incomplete cardiovascular risk assessment, limited use of validated alcohol screening tools, insufficient longitudinal monitoring, and suboptimal coordination with primary care. These findings suggest that, despite increasing awareness of MASLD, substantial gaps persist in the implementation of clinical recommendations in routine practice. These gaps may be driven by factors such as time constraints, perceived complexity of guidelines, limited institutional support, and unequal access to resources.

Our results are consistent with findings from several international surveys that have evaluated clinical practices related to MASLD or non-alcoholic fatty liver disease (NAFLD). The international POP-NEXT study reported that although most specialists recognize the prognostic importance of fibrosis and are familiar with non-invasive diagnostic strategies, the systematic application of guideline-recommended algorithms remains inconsistent across clinical settings [13]. Similarly, surveys conducted among physicians in North America and other regions have identified substantial educational and implementation gaps in the management of MASLD and metabolic dysfunction-associated steatohepatitis [18,19]. These studies consistently demonstrate that awareness of MASLD does not necessarily translate into systematic implementation of diagnostic pathways.

Our results also align with studies conducted in other healthcare systems showing variability in the use of non-invasive biomarkers and elastography. In the POP-NEXT survey, fewer than half of respondents reported systematic use of structured fibrosis risk algorithms in clinical practice [13]. By contrast, in our study, non-invasive fibrosis biomarkers such as FIB-4 were widely used, suggesting increasing adoption of these tools in Spanish hepatology practice. Nevertheless, the limited use of steatosis biomarkers and the heterogeneous application of screening strategies indicate that diagnostic pathways remain incompletely standardized. Within Spain, our findings complement recent evidence from primary care. A national survey among Spanish primary care physicians reported limited use of fibrosis biomarkers and a relatively low perception of the clinical relevance of MASLD [15]. Taken together, these results indicate that gaps exist across multiple levels of the healthcare system. Even among gastroenterologists—who represent the reference specialists for liver disease—important challenges remain in the systematic implementation of diagnostic and management strategies. The limited use of validated screening tools for alcohol intake may increase the risk of underreporting and misclassification, particularly given the overlap between MASLD and alcohol-related liver disease. This highlights the need for more standardized approaches to alcohol assessment in clinical practice.

A particularly relevant concept emerging from our findings is that of diagnostic opportunity loss. MASLD frequently remains asymptomatic until advanced stages of fibrosis develop, which contributes to delayed diagnosis and reduces the potential for early intervention [20,21]. Given that cardiovascular disease represents the leading cause of death among patients with MASLD [8], the relatively low proportion of specialists reporting systematic cardiovascular risk assessment in our study is concerning. Although this survey did not specifically evaluate the use of newer composite algorithms such as the FAST score, which integrate elastography and biochemical parameters to improve risk stratification, future studies should assess their level of implementation among specialists. In recent years, international experts have proposed the strategic objective of doubling the diagnosis rate of patients with at-risk MASH, defined as MASH with significant fibrosis. Achieving this goal requires widespread adoption of non-invasive screening algorithms, stronger integration of diagnostic pathways in primary care, and broader involvement of non-hepatology specialties [22]. Our findings support the relevance of this initiative. The absence of systematic longitudinal evaluation and the incomplete integration of metabolic risk assessment observed in our survey suggest that a substantial number of patients with advanced disease may remain undiagnosed within current clinical pathways.

Beyond clinical implications, these gaps also have important economic consequences. MASLD has been identified as a major driver of healthcare expenditures associated with liver and metabolic diseases worldwide [1]. Strengthening early detection strategies and improving coordination between primary care and specialist services may therefore reduce not only disease-related complications but also long-term healthcare costs. This perspective aligns with international proposals advocating for the recognition of MASLD as a global public health priority requiring coordinated policy responses [23].

Other relevant findings from our survey include the limited use of steatosis biomarkers and the low frequency of waist circumference measurement, both of which are useful indicators for assessing metabolic risk. The low rate of waist circumference measurement, despite its recognized relevance in MASLD, likely reflects several factors. First, routine clinical practice in gastroenterology settings often prioritizes laboratory and imaging parameters over anthropometric measurements. Second, time constraints in outpatient clinics may limit the systematic collection of such data. Third, waist circumference assessment is traditionally more embedded in primary care or endocrinology settings rather than in specialist practice. Similar limitations have been described in other surveys evaluating the management of MASLD and cardiovascular risk among digestive disease specialists [24]. Differences according to professional profile and workplace setting were also observed. Hepatologists, younger specialists, and physicians working in larger hospitals reported greater adherence to guidelines and more frequent use of biomarkers and elastography. Conversely, general gastroenterologists and professionals working in smaller centers reported more limited access to diagnostic tools. These findings suggest that structural factors—including institutional resources, subspecialty training, and access to technology—may play a significant role in shaping MASLD management practices.

Improving training and strengthening collaboration between healthcare levels therefore emerge as key priorities. Continuous education initiatives, particularly focused on non-invasive diagnostic tools and longitudinal disease monitoring, could substantially improve clinical practice. At the same time, strengthening coordination with primary care remains essential. In our survey, most respondents reported limited availability of shared clinical protocols and joint clinical meetings with primary care physicians. Similar barriers have been reported in other European healthcare systems, where insufficient integration between primary care and specialist services has been identified as a major obstacle to effective MASLD management [25]. Emerging digital technologies, implementation of structures referral algorithms, joint clinical protocols, multidisciplinary collaboration and targeted educational programs may offer new opportunities to address these challenges. Artificial intelligence-based risk stratification tools, telemedicine strategies, and digital care pathways are increasingly being explored in hepatology and may help optimize screening, monitoring, and coordination of care in MASLD [26,27].

Finally, recent therapeutic advances further reinforce the importance of addressing these gaps. The approval of resmetirom for patients with MASH and fibrosis by the U.S. Food and Drug Administration represents the first disease-specific pharmacological therapy targeting this condition [9]. In parallel, metabolic therapies such as semaglutide have demonstrated promising efficacy in patients with metabolic dysfunction-associated steatohepatitis [10]. With the emergence of disease-modifying therapies for MASH, optimizing early identification, risk stratification, and multidisciplinary care pathways becomes increasingly critical.

In conclusion, this nationwide survey provides a comprehensive overview of MASLD management among gastroenterologists in Spain. Although awareness of the disease is high and non-invasive diagnostic tools are widely available, important gaps remain in cardiovascular screening, alcohol assessment, guideline implementation, and longitudinal monitoring. Insufficient coordination with primary care further contributes to delayed diagnosis and missed therapeutic opportunities in patients with at-risk MASH. These findings are particularly relevant in the context of the evolving paradigm of integrated hepatometabolic care, which emphasizes the need for coordinated management of liver and cardiometabolic risk. Addressing these challenges will require strengthened training programs, equitable access to diagnostic technologies, systematic cardiometabolic screening strategies, and the development of integrated multidisciplinary care pathways across the healthcare system.

## Figures and Tables

**Table 1:** Sociodemographic and professional characteristics of respondents.

Variable	Value
Sex	Women: 230 (56.4%)
	Men: 173 (42.4%)
Age	<30 years old: 42 (10.3%)
	30–40 years old: 148 (36.3%)
	40–50 years old: 88 (21.6%)
	>50 years old: 130 (31.9%)
Average experience	<5 years: 99 (24.3%)
	5–10 years: 87 (21.3%)
	10–20 years: 87 (21.3%)
	>20 years: 135 (33.1%)
Type of hospital	Public: 324 (79.4%)
	Private: 48 (11.8%)
	Mixed: 32 (7.8%)
Size of hospital	>750 beds: 119 (29.2%)
	250–750 beds: 175 (42.9%)
	<250 beds: 78 (19.1%)
Practice area	General gastroenterologist: 220 (51.3%)
	Hepatologists: 189 (44.1%)
	Endoscopists: 190 (44.3%)

**Table 2:** Use of serum biomarkers for fibrosis and steatosis, and imaging techniques.

Variable	Value
Biomarkers of fibrosis	FIB-4: 258 (63.2%)
	NAFLD Fibrosis Score: 153 (37.5%)
	APRI: 150 (36.8%)
	HEPAMET fibrosis score: 82 (20.1%)
	Forns: 37 (9.1%)
	ELF: 12 (2.9%)
	Fibrotest: 11 (2.7%)
	OWLiver: 26 (6.4%)
	None: 20 (4.9%)
Biomarkers of steatosis	FLI: 92 (22.6%)
	HSI: 36 (8.8%)
	OWLiver: 24 (5.9%)
	None: 162 (39.7%)
Imaging techniques	FibroScan: 249 (61.0%)
	Ultrasound: 128 (31.4%)
	RM: 23 (5.6%)
	None: 16 (3.9%)

## Data Availability

The data presented in this study are available on request from the corresponding author due to ethical reasons.

## Supplementary Materials

The following supporting information can be downloaded at: https://www.mdpi.com/article/10.3390/jcm15093259/s1, Supplementary File S1: MASLD management in Spain: a nationwide survey of gastroenterologists highlighting gaps in risk assessment and Primary Care coordination—Questionnaire.

## Abbreviations

The following abbreviations are used in this manuscript:

AEEH	Spanish Association for the Study of the Liver
AUDIT	Alcohol Use Disorders Identification Test
CHERRIES	Checklist for Reporting Results of Internet E-Surveys
EASL	European Association for the Study of the Liver
FLI	Fatty Liver Index
HSI	Hepatic Steatosis Index
MASLD	Metabolic dysfunction-associated steatotic liver disease
MASH	Metabolic dysfunction–associated steatohepatitis
NAFLD	Non-alcoholic fatty liver disease
SD	Standard deviations
SEPD	Spanish Society of Digestive Diseases
STROBE	Strengthening the Reporting of Observational Studies in Epidemiology
