# Supplementary material for: MASLD Management in Spain: A Nationwide Survey of Gastroenterologists Highlighting Gaps in Risk Assessment and Primary Care Coordination
Source: J Clin Med. 2026 Apr 24;15(9):3259. doi: 10.3390/jcm15093259 (PMC13164508; doi:10.3390/jcm15093259)
Supplement: Supplementary file 1 [file jcm-15-03259-s001.zip › jcm-4234591-supplementary.pdf]

MASLD management in Spain: a nationwide survey of gastroenterologists highlighting gaps in risk assessment and Primary Care coordination - Questionnaire

Number	Question
General Data	
1	What is your gender?
2	What is your age?
3	What type of medical center do you work in?
4	Years of experience as a gastroenterologist
5	Please indicate in which autonomous community you work
6	The number of beds in the hospital where you work
7	The primary area of gastroenterology where you work (multiple-choice question)
Questions on Diagnosis and Monitoring	
8	What is the prevalence of hepatic steatosis in your practice?
9	How many patients with metabolic hepatic steatosis do you see on average per month?
10	What approximate percentage of the patients you see have metabolic hepatic steatosis?
11	In what situations do you think metabolic hepatic steatosis should be ruled out? (multiple-choice question)
12	Which of the following factors is associated with the risk of liver mortality in patients with Metabolic Hepatic Steatosis (MHS)? (multiple-choice question)
Knowledge and Use of Methods for the Evaluation of Fibrosis and Steatosis	
13	Are you aware of the biomarkers used for detecting hepatic fibrosis in patients with metabolic hepatic steatosis?
14	Do you use more than one marker to detect hepatic fibrosis in patients with metabolic hepatic steatosis?
15	Are you aware of the biomarkers used for detecting hepatic steatosis in patients with metabolic hepatic steatosis?
16	Do you use elastography to detect hepatic fibrosis in patients with metabolic hepatic steatosis?
17	Do you measure waist circumference in patients with suspected hepatic steatosis?
18	Do you measure weight and height and calculate body mass index (BMI) in patients with suspected hepatic steatosis?
19	When metabolic liver disease is suspected, do you perform a targeted assessment of vascular risk factors?
20	Please indicate which vascular risk factors you specifically assess in this condition (multiple-choice question).

21	Which of the following fibrosis biomarkers are available at your center and do you use for the diagnosis and follow-up of patients with metabolic hepatic steatosis? (multiple-choice question)
22	Which of the following steatosis biomarkers are available at your center and are utilized for the diagnosis and monitoring of patients with metabolic hepatic steatosis? (multiple-choice question)
23	What elastography methods for hepatic fibrosis assessment are available at your center, and which do you use for the diagnosis and monitoring of patients with metabolic hepatic steatosis? (multiple-choice question)
24	Do you have access to hepatic biopsy at your center for the diagnosis and monitoring of patients with metabolic hepatic steatosis?
Knowledge of Guidelines by Digestive Specialists	
25	Are you familiar with the updated definition and classification of metabolic hepatic steatosis?
26	Do you know any clinical guidelines for the diagnosis and monitoring of patients with metabolic hepatic steatosis?
27	Do you follow any clinical guidelines for the diagnosis and monitoring of patients with metabolic hepatic steatosis?
28	If you follow any clinical guidelines for the diagnosis and monitoring of patients with metabolic hepatic steatosis, which one do you use? (multiple-choice question)
Assessment of alcohol consumption	
29	In your opinion, can alcohol play a role in liver damage in patients with metabolic dysfunction–associated steatotic liver disease?
30	Regarding alcohol consumption in patients with suspected metabolic dysfunction–associated steatotic liver disease, do you routinely assess it?
31	If you assess alcohol consumption in these patients, how do you evaluate it? (multiple-choice question)
Relationship with Primary Care	
32	Do you think that primary care physicians are familiar with the updated definition and classification of metabolic hepatic steatosis?
33	Do you believe that primary care physicians are familiar with the use of biomarkers for detecting hepatic fibrosis in patients with Metabolic Associated Steatotic Liver Disease (MASLD)?
34	Do you think that primary care physicians employ these markers to detect hepatic fibrosis in patients with metabolic hepatic steatosis? (multiple-choice question)
35	Do you think that Primary Care physicians use more than one marker to detect liver fibrosis in patients with metabolic dysfunction–associated steatotic liver disease?
36	Do you think that Primary Care physicians are familiar with the use of biomarkers to detect hepatic steatosis in patients with metabolic dysfunction–associated steatotic liver disease?

37	Do you think that access to abdominal ultrasound from Primary Care is adequate?
38	Do you think that access to elastography (FibroScan®) from Primary Care is adequate?
39	Do you think that Primary Care physicians who have access to elastography (FibroScan®) use it appropriately to detect liver fibrosis in patients with metabolic dysfunction–associated steatotic liver disease?
40	How would you describe your or your department's relationship with primary care at present?
41	Are there regular meetings between the two specialties?
42	Do you believe that meetings between Gastroenterology and Primary Care are or would be beneficial for improving care for patients with metabolic hepatic steatosis?
43	Do you think that the management of metabolic dysfunction–associated steatotic liver disease by Primary Care physicians is adequate?
44	What measures do you think could improve collaboration and communication between gastroenterology specialists and Primary Care physicians in the management of metabolic dysfunction–associated steatotic liver disease? (multiple-choice question)
45	Do you or any member of your department participate in any type of educational activity with Primary Care physicians regarding metabolic dysfunction–associated steatotic liver disease?
46	Does your department share any protocols for the diagnosis, referral, and/or management of patients with metabolic dysfunction–associated steatotic liver disease?
47	In your opinion, are the roles and responsibilities between gastroenterology services and Primary Care centers clearly defined?
48	In the management of patients with metabolic dysfunction–associated steatotic liver disease, do you provide recommendations or guidance to Primary Care physicians regarding patient management?
49	Do you consider that collaboration and communication between gastroenterology specialists and Primary Care physicians in the management of metabolic dysfunction–associated steatotic liver disease should be improved?
50	In your opinion, do Primary Care physicians adequately assess alcohol consumption in patients with suspected metabolic dysfunction–associated steatotic liver disease?
51	If you assess alcohol consumption in these patients, how do you evaluate it? (multiple-choice question)
52	Do you think that Primary Care physicians assess alcohol consumption better, worse, or similarly compared to gastroenterology specialists?
53	What measures do you think could improve collaboration and communication between gastroenterology specialists and Primary Care

	physicians in the management of metabolic dysfunction–associated steatotic liver disease? (open-ended question)
